# Assessing Gambling Disorder Using Semistructured Interviews or Self-Report? Evaluation of the Structured Clinical Interview for Gambling Disorder Among Swedish Gamblers

**DOI:** 10.1177/10731911221147038

**Published:** 2023-01-21

**Authors:** Olof Molander, Viktor Månsson, Anne H. Berman, Jon E. Grant, Peter Wennberg

**Affiliations:** 1 Karolinska Institutet, Stockholm, Solna, Sweden; 2 Stockholm Region Health Services, Sweden; 3 Uppsala University, Sweden; 4 The University of Chicago, IL, USA; 5 Stockholm University, Sweden; 6 Inland Norway University of Applied Sciences, Lillehammer, Norway

**Keywords:** gambling disorder, diagnosis, SCI-PG, SCI-GD, *DSM-5*, SCID

## Abstract

The Structured Clinical Interview for Gambling Disorder (SCI-GD) has the potential to bridge a diagnostic clinical gap, but psychometric evaluations have been scarce, in particular in relation to self-reported diagnostic criteria. This study analyzed existing data, including Swedish gamblers (*N* = 204) from treatment- and help-seeking contexts, self-help groups, and the general population, who were interviewed with the SCI-GD and completed self-report measures. The results indicated that fewer individuals fulfilled the diagnostic criteria for gambling disorder (GD) with the SCI-GD (*n* = 110, 54%), compared to a self-report *Diagnostic and Statistical Manual of Mental Disorders:5th Edition* (*DSM-5*) questionnaire on GD (*n* = 145, 71%; *p* < .001). Agreement between interviews and self-reported criteria was generally low (Fleiss kappa range: 0.31–0.52; *r* range: 0.35–0.55). A Rasch analysis showed that specific diagnostic criteria varied in difficulty, indicating a general pattern of higher item difficulty for the SCI-GD compared to self-reported *DSM-5* criteria. Both the SCI-GD and the self-reported *DSM-5* criteria performed well in terms of internal consistency, convergent, and discriminant validity. We conclude that the SCI-GD is a reliable and valid diagnostic tool to assess GD among individuals with various gambling behavior patterns. Further research-related and clinical implications are discussed.

Addictive behaviors encompass alcohol and other substances, gambling, gaming, sexual behaviors, shopping and other behaviors that yield pleasure at low levels of activity but are frequently associated with severe negative consequences at higher levels of activity. Three of these behaviors are diagnosable as psychiatric disorders: alcohol, substances, and gambling. Gambling was long defined as an impulsive disorder, but in 2013, a landmark step was taken to classify gambling disorder (GD) as an addictive disorder, together with alcohol use disorder (AUD) and substance use disorder (SUD) in the *Diagnostic and Statistical Manual of Mental Disorders: 5th edition* (*DSM-5*; American Psychiatric Association, 2013). GD is diagnosed according to nine criteria describing negative consequences during the past 12 months (see Table 1).

**Table 1.** Gambling Disorder According to DSM-5.

Criteria		Description
A1	Tolerance	Needing to gamble with increased amounts of money to achieve the desired excitement.
A2	Abstinence	Feeling restless or irritable when attempting to cut down or stop gambling.
A3	Loss of control	Having made repeated unsuccessful efforts to control, cut back or stop gambling.
A4	Preoccupation	Having persistent thoughts of reliving past gambling experiences, planning the next gamble opportunity, or thinking of ways to obtain money for gambling again.
A5	Escape	Frequent gambling when distressed or experiencing negative emotions.
A6	Chasing losses	After losing money gambling, often returning another day to gamble in order to get even.
A7	Lying	Lying to conceal the scope of one’s involvement in gambling.
A8	Jeopardized relationships, work, and/or education	Having jeopardized personal relationships, work, and/or educational opportunities due to gambling.
A9	Relies on others	Relying financially on others to provide money to relieve desperate gambling-related financial situations.
B1	Exclusion criterion: gambling due to manic episodes	Gambling exclusively during manic episodes.

*Note. DSM*-5 = the *Diagnostic and Statistical Manual of Mental Disorders: 5th Edition* (American Psychiatric Association, 2013).

If an individual meets at least 4 of 9 inclusion criteria (A) during the past 12 months (and not the exclusion criterion [B]), a GD diagnosis is established. For addictive behaviors in the *DSM-5*, a continuum is used to classify individuals according to three levels of severity. For GD, individuals meeting 4 to 5 criteria are diagnosed with mild GD, 6 to 7 criteria are labeled as moderate GD, and 8 to 9 criteria yield a diagnosis of severe GD.

Little has been reported about the GD diagnosis, for example, concerning diagnostic prevalence, prevalence of severity levels (Molander et al., 2021), or how specific criteria correspond to clinical severity. GD can be assessed with both self-report measures (see for example Molander et al., 2021) and diagnostic interviews. The Structured Clinical Interviews for Pathological Gambling/Gambling Disorder (SCI-PG/SCI-GD) were developed by Grant et al. (2004) using the same procedure of assessment as the Structured Clinical Interview (SCID; First et al., 2016). The first version of the interview was named SCI-PG based on the *Diagnostic and Statistical Manual of Mental Disorders* (4th ed.; *DSM-IV*; American Psychiatric Association, 1994) nomenclature, while the second version, termed SCI-GD, is an adaptation for the GD diagnosis according to the later *DSM-5*. The SCI-GD is a clinician-administered structured diagnostic interview which has been used as a gold standard assessment procedure in gambling research (see, for example, Campos et al., 2016; Chamberlain et al., 2017; Chamberlain & Grant, 2018a, 2018b; Grant et al., 2005, 2015; Månsson et al., 2022; Molander et al., 2021; Petry et al., 2006; Schreiber et al., 2012). The SCI-GD has the potential to bridge an important diagnostic clinical gap. In a recent register study, Håkansson et al. (2018) concluded that very few of the expected GD cases in the general population had been diagnosed with GD in the Swedish health care system. The SCI-GD has shown excellent psychometric properties in an U.S. sample of treatment-seeking gamblers (Grant et al., 2004), but apart from that, psychometric evaluations of the SCI-GD have been lacking.

Self-report measures and diagnostic interviews are often combined in clinical and research settings, for example, using stepwise assessment models (see Berman et al., 2022) where self-report measures are used to screen for case identification as well as further exploration of the extent of the problem, and followed by more extensive, resource-intensive diagnostic interviews as needed to clarify the clinical conceptualization. However, according to our knowledge, the relationship between self-report measures and diagnostic interviews has not been studied for GD, and it is not clear whether *DSM-5* criteria might show disparate diagnostic outcomes when assessed with these two different methods and whether the diagnostic criteria might perform differently when assessed via self-report or diagnostic interview, respectively.

This study has two aims. The first aim is to evaluate the psychometric properties of the Swedish version of the SCI-GD in terms of reliability, validity, item fit, item difficulty, and number of diagnostic strata. The second aim of the study is to compare psychometric performance, that is, reliability, validity, classification, item coherence, item fit, item difficulty, and number of diagnostic strata, for the SCI-GD in relation to self-reported GD criteria, to identify potential differences and discuss clinical implications.

## Method

### Participants and Procedure

This study analyzed data from a previous psychometric study evaluating the Gambling Disorder Identification Test (GDIT: see Molander et al., 2021). Briefly, the psychometric study recruited gamblers (*N* = 603) from treatment- and help-seeking contexts, self-help groups and the general population. Consenting participants completed self-report measures in an online survey. A sub-sample (*n* = 204) was interviewed with the SCI-GD (Grant et al., 2004). The total sample analyzed in this study consists of this subsample; see Table 2 for participant characteristics. At the start of data collection in the psychometric study (Molander et al., 2021), all participants who completed the online survey were contacted for a SCI-GD interview. Later during the study, only participants with higher gambling severity in self-report scores were prioritized to partake in an SCI-GD interview to obtain a sufficiently heterogeneous sample of participants in each GD severity level. Participants who agreed to be interviewed were reimbursed with two movie vouchers. The psychometric study was approved by the Regional Ethics Board of Stockholm, Sweden (ref. no. 2017/1479-31), and all participants provided informed consent for study participation and publication of results, including the results published in this study.

**Table 2.** Participant Characteristics Across Samples.

Characteristics	Gambling cohort				Gender		Total
	Recreational (*n* = 43)	Help-seeking (*n* = 79)	Self-help groups (*n* = 31)	Treatment seeking (*n* = 51)	Men (*n* = 152)	Women (*n* = 52)	Total (*N* = 204)
Age, *M* (*SD*)	33.7 (9.9)	37.9 (14.4)	41.2 (9.1)	36.6 (10.2)	36 (12.1)	40.9 (11)	37.2 (12.0)
Source of income (%)							
Employed	51	67	71	76	68	62	67
Studies	35	10	6	4	15	8	13
Other	14	23	23	20	16	31	20
Highest level of education (%)							
University	56	33	19	27	35	33	34
High school	35	49	68	63	53	52	52
Junior high school	7	13	10	6	9	10	9
Civil status (%)							
Cohabiting	58	57	68	37	55	50	54
Children	40	51	77	55	51	60	53
Gambling characteristics (%)							
Gambling debts	23	53	97	92	57	81	63
Gambling types							
Casino online	44	62	71	84	61	79	65
Casino land-based	28	9	16	14	18	8	15
Sport games online	49	29	39	43	49	6	38
Sport games venue	12	10	19	18	18	0	14
Poker online	26	8	13	18	20	0	15
Poker club	9	5	3	6	8	0	6
EGM	2	11	26	8	10	13	11
Number games	9	13	10	8	13	2	10
Lotteries	35	19	10	18	22	17	21
Horse betting	23	19	26	20	23	15	21
Bingo	2	9	13	10	7	13	8
Other	16	10	6	4	12	2	9

*Note*. Participants were able to report several gambling types. EGM = electronic gambling machine; SCI-GD = Structured Clinical Interview for Gambling Disorder (Grant et al., 2004).

### Raters

The SCI-GD interviews were conducted by telephone, which is a reliable procedure for psychiatric assessment (Cantwell et al., 1997). Interviewers were four licensed psychologists and four advanced clinical psychology students at the MSc level (mean *n* interviews per rater: 35; range: 1–65 interviews per rater). Prior to conducting the interviews, all interviewers participated in a workshop for diagnostic assessment of GD, held by author O.M. The workshop included an assessment of inter-rater agreement, whereby each of the eight interviewers rated a calibration interview consisting of a prerecorded video of a fictitious gambler interviewed with the SCI-GD.

### Measures

#### The SCI-GD

The SCI-GD (Grant et al., 2004) is a clinician-administered structured interview assessing the *DSM-5* GD diagnostic inclusion criteria (part A), and including the exclusion criterion (part B) of “gambling due to manic episode,” as well as assessment of diagnostic severity. The SCI-GD follows the SCID (First et al., 2016) interview format, meaning that the clinician asks as many questions as necessary, to be able to assess whether or not a criterion has been fulfilled. The first SCI-GD section includes a series of questions regarding behavioral descriptions, for example, current gambling frequency, gambling expenditures, and the most intense gambling period in the respondent’s lifetime. In the second SCI-GD section, each GD criterion is assessed via an initial item, and several optional follow-up items. For instance, Concerning A4_Preoccupation_: “How often do you think about gambling?,” with follow-up questions such as “How much do you think about past gambling experiences?” and “How often do you imagine or plan future gambling?.” Each criterion is coded as “? = Inadequate information,” “1 = *absent or false*,” “2 = *subthreshold*,” or “3 = *threshold or true*.” This section ends with questions on the exclusion criterion. In the third and final section, the diagnostic assessment is summarized. For a diagnosis of GD, at least 4 criteria (A) must be present (coded as “3 = *threshold or true*”) during the past 12 months, and the exclusion criteria (B) may not be fulfilled. As noted above, individuals meeting 4 to 5 criteria are diagnosed with mild GD, fulfillment of 6 to 7 criteria is labeled as moderate GD, and 8 to 9 fulfilled criteria yield a diagnosis of severe GD. Furthermore, GD can be specified as episodic/persistent or in early or sustained remission.

Psychometric properties of the SCI-GD have been evaluated in a sample of treatment seeking gamblers (*N* = 72) (Grant et al., 2004). Test–retest and inter-rater reliability were excellent (*r* = 0.97 and kappa = 1.00, respectively). Concurrent validity was examined in relation to the South Oaks Gambling Screen (SOGS; Lesieur & Blume, 1987) and the Yale Brown Obsessive Compulsive Scale adapted for Pathological Gambling (PG-YBOCS; Pallanti et al., 2005; *r* = 0.78 and *r* = 0.37, respectively). In terms of discriminant validity, the SCI-GD showed weak correlations with the Hamilton Depression and Anxiety Rating Scales (HAM-D and HAM-A; Hamilton, 1959, 1960; *r* = 0.19 and *r* = 0.23, respectively). Finally, classifications were evaluated in relation to a longitudinal course of illness. Here the SCI-GD showed high sensitivity (0.88) and perfect specificity (1.00).

The Swedish SCI-GD translation was conducted using a back-translation procedure. In the first step, the original English SCI-GD was collaboratively translated into Swedish, by authors O.M. and V.M. The Swedish SCI-GD translation was also modified to assess each GD criterion during the previous 12 months, in addition to the most intense lifetime gambling period. In the second step, a native English speaker (author A.H.B.), naive to the original SCI-GD interview, translated the Swedish version back to English. Following additional language modifications, the English back-translation was approved by the developer of the instrument (author J.E.G.). The final version of the Swedish SCI-GD interview is free for use in research and clinical settings (see Supplementary Material 1).

#### Self-Reported Criteria for GD

GD was also assessed using a newly developed 9-item self-report questionnaire with dichotomous response alternatives (yes/no) for each GD criterion. For instance, the question regarding Preoccupation (A4) was “Do you constantly think about gambling? For example, are you busy thinking about previous gambling experiences, planning your next gambling opportunity, or thinking about how you can raise money to gamble with?” Assessment of the exclusion criterion (B) was not included in the self-report questionnaire. The self-report *DSM-5* questionnaire was chosen as an SCI-GD comparator in this study, as the item content of the measure was mirroring the *DSM-5* criteria included in the SCI-GD interview. This measure was administered online.

#### Self-Report Measures

Additional measures in the study included the GDIT (Molander et al., 2021; www.gditscale.com), the Problem and Pathological Gambling Measure (PPGM; Williams & Volberg, 2013), the Patient Health Questionnaire 9-item scale measuring depression (PHQ-9; Kroenke et al., 2001), the Generalized Anxiety Disorder 7-item scale measuring anxiety (GAD-7; Spitzer et al., 2006), and the Mood Disorder Questionnaire, a screener for bipolar disorder and manic symptoms (MDQ; Hirschfeld et al., 2000). The presence of gambling debts was measured using a dichotomous (yes/no) question phrased: “Do you have debts due to gambling?.” All these self-report measures were administered online.

### Statistical Analysis and Data Preparation

An a priori statistical plan was published via OSF Registries (DOI: 10.17605/OSF.IO/U8BDX). A network analysis, listed as a potential analysis in the statistical plan, was not performed due to power reasons and a decision that this analysis was outside the scope of this article.

The Welch two-sample *t*-test was used to compare GD classification of SCI-GD with self-reported *DSM-5* criteria, not originally specified in the statistical plan. Also, in addition to Cronbach’s α, McDonald’s ω_*h*_ (McDonald, 1999) was estimated as a complementary reliability measure. Seven participants had missing data (>3%) for all items in the self-report *DSM-5* questionnaire, as well as the GDIT, PPGM, PHQ-9, GAD-7, and MDQ. These missing items were handled using multivariate imputation via chained equations (predictive mean matching, five data sets, 70–126 iterations per imputation; Enders et al., 2016).

Rasch analysis was selected because it offers several advantages in relation to the study aims. It enables evaluation of specific diagnostic criteria across a severity continuum, and assessment and comparison of how criteria respond to different levels of symptom severity (e.g., item difficulty). It also provides the opportunity of testing how many strata a measure reliably can be divided into, an important factor in relation to the proposed levels of GD severity in the *DSM-5*. In a Rasch analysis, a person-separation reliability estimate of >70 indicates one stratum, an estimate of >80 indicates two strata, while estimates of >90 and >94 indicate three and four strata, respectively (Wright & Masters, 1982). A Rasch analysis could also ideally establish that an instrument lies on a data level that allows advanced statistical analyses (i.e., parametric analyses), a factor that is important from a research perspective. A Rasch model assumes that a single latent variable is sufficient to explain most of the variation in item responses (i.e., unidimensionality), an assumption that can be tested by estimating the proportion of variance in responses attributable to item and person locations on the primary latent variable and by principal component analyses of standardized residual correlations (see Model fit below; Wind & Hua, 2021). The Rasch model also assumes equal discrimination across items. Two-item fit measures to test this assumption are infit (inlier-sensitive or information-weighted fit) and outfit (outlier-sensitive fit). Infit and outfit item estimates between <0.50 (i.e., overfit) and >1.50 (i.e., underfit) indicate equal discrimination across items and can be considered productive for measurement (Linacre, 2002; Wind & Hua, 2021). The Rasch analysis in this study used a dichotomous model for the GD criteria, assessed via SCI-GD interviews and self-report, estimated as dichotomized 1 or 0 variables.

Comparisons were made between the SCI-GD interviews and self-reported GD criteria in the total sample (*N* = 204). Pearson’s *r* and Tetrachoric correlations (assuming that the latent variables are continuous) were used to compare assessments via SCI-GD and self-report, for each GD criterion. Fleiss’ kappa for multiple raters and categorical variables (Fleiss, 1971) was used to compare assessments between the SCI-GD and the self-report questionnaire, for each GD criterion (dichotomous yes/no variables), as well as GD severity (categorical variables indiating no, mild, moderate, or severe GD). For the SCI-GD inter-rater calibration interview, percentages of concordance between the assessors (*n =* 8) were estimated for all GD criteria (categorical yes or no variables).

## Results

### Psychometric Properties of the SCI-GD

#### Reliability

The inter-rater calibration SCI-GD interview had perfect (100%) inter-rater agreements (*n* raters=8) for symptom severity and all GD criteria except for A6_Chasing losses_ (88% agreement) and A9_Relies financially on others_ (63% agreement). Regarding internal consistency of the SCI-GD, estimates of Cronbach’s α^a^ and McDonald ω_h_ were very good in the total sample (α=0.86 and ω_h_=0.90, respectively), and acceptable to very good across sub-samples (range 0.80 to 0.95) (see Table 3).

**Table 3.** Internal Consistency Across Samples.

Gambling disorder	Gambling cohort				Gender		Total
	Recreational (*n* = 43)	Help-seeking (*n* = 79)	Self-help groups (*n* = 31)	Treatment seeking (*n* = 51)	Men (*n* = 152)	Women (*n* = 52)	Total (*N* = 204)
Cronbach’s α^a^							
SCI-GD	0.80	0.86	0.90	0.82	0.85	0.87	0.86
Self-reported *DSM-5* criteria	0.80	0.90	0.82	0.70	0.86	0.85	0.86
McDonald ω_h_^b^							
SCI-GD	0.87	0.91	0.95	0.88	0.89	0.92	0.90
Self-reported *DSM-5* criteria	0.86	0.93	0.90	0.83	0.90	0.90	0.90

*Note.* SCI-GD = Structured Clinical Interview for Gambling Disorder (Grant et al., 2004); *DSM*-5 = *Diagnostic and Statistical Manual of Mental Disorders: 5th Edition* (American Psychiatric Association, 2013).

a Raw alpha. ^b^Total Omega.

#### Validity

In terms of convergent and discriminant validity, the SCI-GD showed moderate to strong positive correlations with self-report measures assessing problem gambling and GD (i.e., the PPGM and the GDIT), and weak positive correlations with having gambling debts as well as measures assessing depression and anxiety (i.e., the PHQ-9 and the GAD-7) (see Table 4 for specific estimates).

**Table 4.** Convergent and Discriminant Validity Across Samples.

Gambling disorder	Gambling cohort				Gender		Total
	Recreational (*n* = 43)	Help-seeking (*n* = 79)	Self-help groups (*n* = 31)	Treatment seeking (*n* = 51)	Men (*n* = 152)	Women (*n* = 52)	Total (*N* = 204)
Convergent validity							
PPGM							
SCI-GD	0.78	0.79	0.83	0.73	0.76	0.90	0.80
Self-reported *DSM-5* criteria	0.80	0.92	0.31	0.61	0.77	0.74	0.77
GDIT							
SCI-GD	0.67	0.76	0.71	0.66	0.71	0.80	0.75
Self-reported *DSM-5* criteria	0.77	0.87	0.45	0.57	0.77	0.70	0.75
Gambling debts							
SCI-GD	0.44	0.60	-0.11	0.25	0.46	0.46	0.48
Self-reported *DSM-5* criteria	0.50	0.64	-0.08	0.10	0.57	0.55	0.57
Discriminant validity							
PHQ-9							
SCI-GD	0.10	0.60	0.49	0.27	0.36	0.62	0.45
Self-reported *DSM-5* criteria	0.80	0.92	0.31	0.61	0.77	0.74	0.77
GAD-7							
SCI-GD	0.07	0.60	0.34	0.33	0.32	0.58	0.41
Self-reported *DSM-5* criteria	0.11	0.71	0.36	0.33	0.41	0.55	0.46

*Note*. Convergent and divergent validity was estimated using Pearson correlation between measure scores, having gambling debts (dichotomized), and number of fulfilled Gambling Disorder criteria, assessed via SCI-GD interviews or self-report. PPGM = problem and pathological gambling measure (Williams & Volberg, 2013); GD = gambling disorder (American Psychiatric Association, 2013); SCI-GD = Structured Clinical Interview for Gambling Disorder (Grant et al., 2004); *DSM*-5 = Diagnostic and Statistical Manual of Mental Disorders: 5th Edition (American Psychiatric Association, 2013); GDIT = Gambling Disorder Identification Test (Molander et al., 2021); PHQ-9 = Patient Health Questionnaire (Kroenke et al., 2001); GAD-7 = Generalized Anxiety Disorder 7-item scale (Spitzer et al., 2006).

#### Rasch Analysis

##### Model Fit

After Rasch models were fitted for SCI-GD and self-reported *DSM-5* criteria, model fit measures were investigated. For both the SCI-GD and self-reported *DSM-5* criteria, the proportion of variance in responses attributable to item and person locations on the primary latent variable in the Rasch models was above the critical value of 20% (33% and 37%, respectively), which indicated unidimensionality (Reckase, 1979). In addition, principal component analyses of standardized residual correlations were performed. For both the SCI-GD and self-reported *DSM-5* criteria, eigenvalues were below 2.00, suggesting that the correlations among the model residuals primarily reflect randomness, and indicating unidimensionality (Wind & Hua, 2021). No *DSM-5* GD criterion showed under- or overfit in the Rasch analyses, indicating equal discrimination across items.

##### Item Difficulty and Number of Diagnostic Strata

SCI-GD item difficulty was estimated across a severity continuum (difficulty range: −0.50 to 1.28). The criteria with the lowest item difficulty were A4_Preoccupation_ and A6_Chasing losses_, while A1_Tolerance_ and A9_Relies on others_ reflected the highest item difficulty. Person separation reliability showed that the SCI-GD-assessed *DSM-5* criteria could reliably be divided into two strata (Wright & Masters, 1982; see Table 5).

**Table 5.** Rasch Analysis of Gambling Disorder, Assessed Via SCI-GD Interviews or Self-Reported DSM-5 Criteria (N = 204).

*DSM-5* criteria		Item difficulty (*SE*)			Infit		Outfit	
		SCI-GD	Self-reported	Difficulty delta^a^	SCI-GD	Self-reported	SCI-GD	Self-reported
A1	Tolerance	1.20 (0.20)	0.85 (0.18)	0.35	1.11	1.11	1.01	1.16
A2	Abstinence	0.60 (0.19)	−0.61 (0.19)	1.21	1.17	0.82	1.30	0.67
A3	Loss of control	0.11 (0.19)	−1.77 (0.22)	1.88	0.93	0.83	0.97	0.86
A4	Preoccupation	−0.50 (0.20)	0.68 (0.18)	−1.18	0.88	1.05	0.71	0.95
A5	Escape	0.94 (0.19)	−0.79 (0.19)	1.73	1.13	1.06	1.18	1.13
A6	Chasing losses	−0.39 (0.20)	−1.29 (0.20)	0.90	0.72	0.71	0.50	0.50
A7	Lying	0.41 (0.19)	−1.46 (0.21)	1.87	0.85	0.90	0.71	0.83
A8	Jeopardized relationships, work and/or education	0.79 (0.19)	0.32 (0.18)	0.47	1.06	1.25	1.10	1.25
A9	Relies financially on others	1.28 (0.20)	1.05 (0.19)	0.23	0.98	1.09	0.93	1.08
EAP reliability		0.84	0.83					
Underfit				>1.50	0	0	0	0
Overfit				<0.50	0	0	0	0
Person separation reliability (*n* strata)		0.70 (2)	0.72 (2)					

*Note. DSM*-5 = *Diagnostic and Statistical Manual of Mental Disorders: 5th Edition* (American Psychiatric Association, 2013); EAP = Expected A Posteriori; SCI-GD = Structured Clinical Interview for Gambling Disorder (Grant et al., 2004).

a Presented as item difficulty of the SCI-GD minus item difficulty of self-reported *DSM*-5 criteria.

### SCI-GD Compared to Self-Reported GD Criteria

#### Reliability and Validity

The SCI-GD and self-reported *DSM-5* criteria displayed roughly equal estimates of internal consistency and convergent and discriminant validity in the total sample as well as across sub-samples (see Tables 3 and 4).

#### Classification

In the total sample, significantly fewer participants were classified above the threshold (≥4 of 9 criteria) for Any GD with the SCI-GD (*n* = 110, 54%), compared to self-reported *DSM-5* criteria (*n* = 146, 72%; *p <* .001). Descriptive statistics indicated a similar pattern across subsamples (see Table 6).

**Table 6.** Gambling Disorder, Endorsed DSM-5 Criteria by the SCI-GD Diagnostic Interview and Self-Report Questionnaire (N = 204).

Gambling disorder, criteria, and classification		SCI-GD, *n* (%)	Self-reported, *n* (%)
A1	Tolerance	73 (36%)	80 (39%)
A2	Abstinence	89 (44%)	123 (60%)
A3	Loss of control	102 (50%)	152 (75%)
A4	Preoccupation	118 (58%)	85 (42%)
A5	Escape	80 (39%)	128 (63%)
A6	Chasing losses	115 (56%)	141 (69%)
A7	Lying	94 (46%)	145 (71%)
A8	Jeopardized relationships, work and/or education	84 (41%)	96 (47%)
A9	Relies on others	71 (35%)	74 (36%)
Gambling disorder, severity, *n* (%)			
No gambling disorder		94 (46%)	58 (28%)
Any gambling disorder		110 (54%)	146 (72%)
Mild gambling disorder		33 (16%)	43 (21%)
Moderate gambling disorder		34 (17%)	57 (28%)
Severe gambling disorder		43 (21%)	46 (23%)

*Note. DSM*-5 = *Diagnostic and Statistical Manual of Mental Disorders: 5th Edition* (American Psychiatric Association, 2013); SCI-GD = Structured Clinical Interview for Gambling Disorder (Grant et al., 2004).

Assessment of the *DSM-5* GD exclusion criterion, B1_Gambling due to manic episode_, is included in the SCI-GD but was not assessed in the self-reported *DSM-5* criteria. As a complementary exclusion criterion assessment, the bipolar/manic screening measure MDQ was used. For the SCI-GD interview, no participant in the total sample (*N* = 204) was assessed as gambling due to manic episode. For the MDQ, 28% of the total sample screened positive for bipolar disorder/manic symptoms.

#### Item Coherences and Item Difficulties

The comparisons between the SCI-GD assessed and self-reported *DSM-5* criteria showed fair to moderate kappa agreement (range: 0.31–0.52), weak to moderate Pearson’s *r* correlations (range: 0.35–0.55), and stronger Tetrachoric correlations (range: 0.52–0.80). Overall, the criterion with the lowest coherence between SCI-GD assessed and self-reported *DSM-5* criteria was A8_Jeopardized relationships, work and/or education_, and the criterion with the highest coherence was A6_Chasing losses_ (see Table 7).

**Table 7.** Comparisons Between Gambling Disorder DSM-5 Criteria (N = 204).

Gambling disorder *DSM-5*		SCI-GD vs self-reported *DSM-5* criteria (*N* = 204)			
		Correlations		Kappa^a^	
		Pearson’s *r*	Tetrachoric	Kappa	95% CI
A1	Tolerance	.41	.60	0.40	[0.27, 0.54]
A2	Abstinence	.43	.65	0.39	[0.25, 0.53]
A3	Loss of control	.49	.80	0.40	[0.26, 0.53]
A4	Preoccupation	.38	.58	0.34	[0.21, 0.48]
A5	Escape	.39	.62	0.31	[0.18, 0.45]
A6	Chasing losses	.55	.78	0.52	[0.38, 0.65]
A7	Lying	.50	.80	0.40	[0.27, 0.54]
A8	Jeopardized relationships, work and/or education	.35	.52	0.34	[0.21, 0.48]
A9	Relies on others	.45	.66	0.44	[0.32, 0.59]
Symptom severity, gambling disorder		—	—	0.37	[0.29, 0.45]

*Note. DSM*-5 = *Diagnostic and Statistical Manual of Mental Disorders: 5th Edition* (American Psychiatric Association, 2013).

a Fleiss kappa for multiple raters and categorical variables (Fleiss, 1971).

In terms of item difficulty scores, all GD criteria showed disparate estimates for the SCI-GD and self-reported *DSM-5* criteria. For some criteria, that is, A9_Relies financially on others_, A1_Tolerance_, and A8_Jeopardized relationships, work and/or education_, differences in item difficulty were comparably small. However, most GD criteria showed relatively large differences in item difficulty, indicating an overall pattern with higher difficulty in the SCI-GD interviews and lower difficulty in the self-reported *DSM-5* criteria. An exception to this pattern was A4_Preoccupation_, which showed a higher item difficulty in the self-reported *DSM-5* criteria. Similar to the SCI-GD, person separation reliability indicated that self-reported *DSM-5* criteria consisted of two strata (Wright & Masters, 1982; see Table 5).

## Discussion

The aims of this study were to evaluate the psychometric properties of the SCI-GD in a Swedish setting and to compare diagnostic assessment through structured interviews in relation to self-report. Each aim is discussed below, including research-related and clinical implications.

### Psychometric Properties of the SCI-GD

The SCI-GD displayed very good internal reliability as well as expected validity estimates in relation to gambling- and nongambling-related measures. These estimates were mainly consistent across subsamples. Furthermore, the SCI-GD inter-rater assessments of symptom severity and specific GD criteria showed perfect agreement, except for two criteria (A9_Relies on others_ and A6_Chasing losses_). Overall, we conclude that the SCI-GD is a reliable and valid diagnostic tool for assessing GD among various Swedish gambling populations, including treatment-seeking patients.

This study is, to our knowledge, the first Rasch analysis of GD criteria assessed according to *DSM-5*. In terms of item difficulty, each SCI-GD criterion was estimated on a severity continuum, showing some resemblance to a previous Rasch study on the National Opinion Research Center *DSM-IV* Screen for Gambling (NODS; a self-report measure based in on the *DSM-IV* criteria; Molde et al., 2010). Item difficulty for specific GD criteria needs to be studied further from a psychometric and epidemiological perspective. One possible interpretation of the results from this study is that GD criteria with lower difficulty (i.e., A4_Preoccupation_ or A6_Chasing losses_) might reflect more immediate gambling-related behavior, while GD criteria with a higher item difficulty (i.e., A1_Tolerance_ or A9_Relies on others_) might constitute more long-term consequences of continuous gambling. When using the SCI-GD in health care settings, clinicians might consider that patient endorsement of some GD criteria might indicate a relatively mild clinical picture while endorsement of other criteria could reflect more severe impairment in addition to GD symptom severity based on the number of criteria fulfilled.

The Rasch analysis of the SCI-GD, as well as of the self-reported *DSM-5* criteria, indicated good fit measures. This indicated that these measurements of the GD diagnosis are on a data level that permits future research use of statistical parametric analyses. We also conclude that GD can be considered as unidimensional, which is in line with the *DSM-5* theoretical conceptualization. However, the Rasch analysis also showed that the GD criteria (both SCI-GD and self-reported *DSM-5* criteria) could reliably be divided into two, rather than four strata. In a study of the previous *DSM-IV* criteria, Strong and Kahler (2007) reported similar item separation reliability issues for pathological gambling (although the *DSM-IV* did not include categories of severity based on a continuum). In another study, which included patients with GD, assessed with SCI-GD and self-report measures, Grant et al. (2017) found difficulties in discriminating between severity levels, that is, moderate and severe GD. Taken together, these empirical findings challenge the proposed levels of severity in the *DSM-5*.

### SCI-GD Compared to Self-Reported GD Criteria

The results showed that when assessed with the SCI-GD diagnostic interview, a substantially smaller proportion of participants (approximately 1 in 5) fulfilled the diagnosis of GD, compared to self-report. Furthermore, although there was high inter-rater reliability in the calibration interview, agreement between the SCI-GD and self-reported *DSM-5* criteria was low. Self-report measures are often used for screening purposes to predict the presence of a diagnosis. In this regard, they are typically designed to identify as many potential individuals as possible in a population, thus prioritizing sensitivity over specificity. Conversely, diagnostic interviews mainly prioritize specificity over sensitivity, to be certain that individuals fulfill the criteria for a specific diagnosis when a treatment plan is conceptualized. Previous research has shown that the SCI-GD has high diagnostic specificity (Grant et al., 2004). For instance, Goodie et al. (2013) evaluated a self-report measure assessing pathological gambling according to *DSM-IV* (the SOGS; Lesieur & Blume, 1987) in relation to the SCI-GD, in a community sample. The results showed false positives for 195 of 353 participants for self-reported pathological gambling (Goodie et al., 2013). The *DSM-5* (American Psychiatric Association, 2013) suggests a severity level based on the number of criteria fulfilled. However, each GD criterion also represents a continuum in itself. This was shown in our study by the higher tetrachoric correlation coefficients, which are based on the assumption that criteria are latent continuous variables. Regarding assessment format, the self-reported *DSM-5* criteria included one single yes/no item per assessed criterion, while the SCI-GD encompasses a range of potential follow-up questions to conclude whether criteria have been fulfilled or not. These are some possible explanations for the difference in GD classification between the measures.

While some GD criteria (i.e., A1_Tolerance_, A8_Jeopardized relationships, work and/or education_, and A9_Relies on others_) showed less differences in difficulty between the SCI-GD and self-reported *DSM-5*, most GD criteria showed differences in the expected direction: when assessed with the SCI-GD (which prioritizes specificity), criteria showed a higher item difficulty than the self-reported *DSM-5*. An exception to this pattern was A4_Preoccupation_, for which the self-reported *DSM-5* criterion had a higher item difficulty the SCI-GD. A possible explanation for this might lie in differences in item formulation. A4_Preoccupation_ was phrased “Do you *constantly* think about gambling?” in the self-reported *DSM-5* compared to the open question in the SCI-GD “How *often* do you think about gambling?.”

The formulation of the GD exclusion criterion in the SCI-GD interviews was a source of confusion for the study raters which resulted in reporting errors for some interviews, as the criterion was phrased as a double negative, and it was difficult to understand how the exclusion criterion should be coded. To address this, the B1 criterion section in the Swedish SCI-GD was revised as a result of this study. The self-reported *DSM-5* criteria did not include any item assessing the GD exclusion criterion. Instead, the MDQ (Hirschfeld et al., 2000) was used as a complementary self-report measure for manic episodes, where 28% of the total sample screened positive, compared to 0% fulfilling the exclusion criterion in the SCI-GD interviews. However, the MDQ might have been a poor comparator for the SCI-GD. The MDQ is a screener for bipolar disorder/manic symptoms using a lifetime timeframe, while GD is assessed during the past 12 months. Furthermore, the MDQ alone might not be a feasible procedure to reliably detect bipolar disorder/manic symptoms in samples with patients with SUDs (see van Zaane et al., 2012). Prevalence estimates of gambling only during manic episodes are scarce, although the proportion of problem gamblers among individuals with bipolar disorder is high (6%–10%; Jones et al., 2015; McIntyre et al., 2007). From a wider perspective, it is paramount to address comorbid psychiatric disorders, such as bipolar disorder, in clinical settings to offer adequate treatment. However, diagnostic caution should be taken so that the GD exclusion criterion is assessed with high sensitivity; otherwise exclusion from needed gambling treatment might result.

Few differences were observed between the SCI-GD and self-reported *DSM-5* in terms of reliability and validity measures. Instead, the assessment differences lay within diagnostic classification, item coherences, and item difficulty. The inclusion of methods beyond classical test theory, such as Rasch analysis, thus constitute a strength of the study. Additional strengths were that a psychometric evaluation of the SCI-GD, a widely used diagnostic interview, was conducted, partly remedying the scarcity of such evaluations. The evaluation included gamblers from several populations, enabling estimates beyond treatment-seeking patients. The study might also be one of the first Rasch studies of GD, and yielded several important findings in relation to diagnostic assessment. In terms of limitations, some measures (i.e., the PHQ-9 [Kroenke et al., 2001], the GAD-7 (Spitzer et al., 2006) and the MDQ [Hirschfeld et al., 2000]), used a different timeframe than the past 12 months used in the SCI-GD. The SCI-GD was not evaluated in relation to another gold standard assessment tool. However, in this regard, it is not clear how to validate a diagnostic interview in itself, that is, what benchmark it could be compared against. Finally, this study did not include an evaluation of SCI-GD assessments of episodic versus persistent GD or early remission versus sustained GD remission, as these assessments were not performed in all interviews.

Future studies should evaluate psychometric properties of the SCI-GD in non-U.S./Swedish gambling samples, and corroborate findings of item difficulty, as well as *DSM-5* severity levels, potentially in relation to network analysis of endorsed GD criteria. Also, care should be taken to ensure that phrasing in the SCI-GD interview is made as concise and clear as possible, to minimize diagnostic errors. Meanwhile, the SCI-GD can be reliably and validly used in clinical settings where specificity is prioritized. In contrast, the self-report *DSM-5* questionnaire could, together with other robust measures of GD-related problems such as the GDIT and the PPGM, serve as part of initial assessment of GD treatment needs, where sensitivity would be prioritized.

## Supplemental Material

sj-docx-1-asm-10.1177_10731911221147038 – Supplemental material for Assessing Gambling Disorder Using Semistructured Interviews or Self-Report? Evaluation of the Structured Clinical Interview for Gambling Disorder Among Swedish Gamblers Click here for additional data file. Supplemental material, sj-docx-1-asm-10.1177_10731911221147038 for Assessing Gambling Disorder Using Semistructured Interviews or Self-Report? Evaluation of the Structured Clinical Interview for Gambling Disorder Among Swedish Gamblers by Olof Molander, Viktor Månsson, Anne H. Berman, Jon E. Grant and Peter Wennberg in Assessment

sj-docx-2-asm-10.1177_10731911221147038 – Supplemental material for Assessing Gambling Disorder Using Semistructured Interviews or Self-Report? Evaluation of the Structured Clinical Interview for Gambling Disorder Among Swedish Gamblers Click here for additional data file. Supplemental material, sj-docx-2-asm-10.1177_10731911221147038 for Assessing Gambling Disorder Using Semistructured Interviews or Self-Report? Evaluation of the Structured Clinical Interview for Gambling Disorder Among Swedish Gamblers by Olof Molander, Viktor Månsson, Anne H. Berman, Jon E. Grant and Peter Wennberg in Assessment

sj-docx-3-asm-10.1177_10731911221147038 – Supplemental material for Assessing Gambling Disorder Using Semistructured Interviews or Self-Report? Evaluation of the Structured Clinical Interview for Gambling Disorder Among Swedish Gamblers Click here for additional data file. Supplemental material, sj-docx-3-asm-10.1177_10731911221147038 for Assessing Gambling Disorder Using Semistructured Interviews or Self-Report? Evaluation of the Structured Clinical Interview for Gambling Disorder Among Swedish Gamblers by Olof Molander, Viktor Månsson, Anne H. Berman, Jon E. Grant and Peter Wennberg in Assessment
