# Supplementary material for: Assessing Gambling Disorder Using Semistructured Interviews or Self-Report? Evaluation of the Structured Clinical Interview for Gambling Disorder Among Swedish Gamblers
Source: Assessment. 2023 Jan 21;30(8):2387–97. doi: 10.1177/10731911221147038 (PMC10623606; doi:10.1177/10731911221147038)
Supplement: sj-docx-2-asm-10.1177_10731911221147038 – Supplemental material for Assessing Gambling Disorder Using Semistructured Interviews or Self-Report? Evaluation of the Structured Clinical Interview for Gambling Disorder Among Swedish Gamblers [file sj-docx-2-asm-10.1177_10731911221147038.docx]

Table 1

Gambling Disorder according to DSM-5

| Criteria | | Description |
| --- | --- | --- |
| A1 | Tolerance | Needing to gamble with increased amounts of money to achieve the desired excitement. |
| A2 | Abstinence | Feeling restless or irritable when attempting to cut down or stop gambling. |
| A3 | Loss of control | Having made repeated unsuccessful efforts to control, cut back or stop gambling. |
| A4 | Preoccupation | Having persistent thoughts of reliving past gambling experiences, planning the next gamble opportunity, or thinking of ways to obtain money for gambling again. |
| A5 | Escape | Frequent gambling when distressed or experiencing negative emotions. |
| A6 | Chasing losses | After losing money gambling, often returning another day to gamble in order to get even. |
| A7 | Lying | Lying to conceal the scope of one’s involvement in gambling. |
| A8 | Jeopardized relationships, work and/or education | Having jeopardized personal relationships, work and/or educational opportunities due to gambling. |
| A9 | Relies on others | Relying financially on others to provide money to relieve desperate gambling-related financial situations. |
| B1 | Exclusion criterion: gambling due to manic episodes | Gambling exclusively during manic episodes. |

DSM-5 = the Diagnostic and Statistical Manual of Mental Disorders–fifth edition [(American Psychiatric Association, 2013)](https://www.zotero.org/google-docs/?richsW)

Table 2

Participant characteristics across samples

|  | Gambling cohort | | | |  | Gender | |  | Total |
| --- | --- | --- | --- | --- | --- | --- | --- | --- | --- |
| Characteristics | Recreational  n=43 | Help-  seeking  n=79 | Self-help groups  n=31 | Treatment seeking  n=51 |  | Men  n=152 | Women  n=52 |  | Total  N=204 |
| Age, M (Sd) | 33.7 (9.9) | 37.9 (14.4) | 41.2 (9.1) | 36.6 (10.2) |  | 36 (12.1) | 40.9 (11) |  | 37.2 (12.0) |
| Source of income (%) |  |  |  |  |  |  |  |  |  |
| Employed | 51 | 67 | 71 | 76 |  | 68 | 62 |  | 67 |
| Studies | 35 | 10 | 6 | 4 |  | 15 | 8 |  | 13 |
| Other | 14 | 23 | 23 | 20 |  | 16 | 31 |  | 20 |
| Highest level of education (%) |  |  |  |  |  |  |  |  |  |
| University | 56 | 33 | 19 | 27 |  | 35 | 33 |  | 34 |
| High school | 35 | 49 | 68 | 63 |  | 53 | 52 |  | 52 |
| Junior high school | 7 | 13 | 10 | 6 |  | 9 | 10 |  | 9 |
| Civil status (%) |  |  |  |  |  |  |  |  |  |
| Cohabiting | 58 | 57 | 68 | 37 |  | 55 | 50 |  | 54 |
| Children | 40 | 51 | 77 | 55 |  | 51 | 60 |  | 53 |
| Gambling characteristics (%) |  |  |  |  |  |  |  |  |  |
| Gambling debts | 23 | 53 | 97 | 92 |  | 57 | 81 |  | 63 |
| Gambling types |  |  |  |  |  |  |  |  |  |
| Casino online | 44 | 62 | 71 | 84 |  | 61 | 79 |  | 65 |
| Casino land-based | 28 | 9 | 16 | 14 |  | 18 | 8 |  | 15 |
| Sport games online | 49 | 29 | 39 | 43 |  | 49 | 6 |  | 38 |
| Sport games venue | 12 | 10 | 19 | 18 |  | 18 | 0 |  | 14 |
| Poker online | 26 | 8 | 13 | 18 |  | 20 | 0 |  | 15 |
| Poker club | 9 | 5 | 3 | 6 |  | 8 | 0 |  | 6 |
| EGM | 2 | 11 | 26 | 8 |  | 10 | 13 |  | 11 |
| Number games | 9 | 13 | 10 | 8 |  | 13 | 2 |  | 10 |
| Lotteries | 35 | 19 | 10 | 18 |  | 22 | 17 |  | 21 |
| Horse betting | 23 | 19 | 26 | 20 |  | 23 | 15 |  | 21 |
| Bingo | 2 | 9 | 13 | 10 |  | 7 | 13 |  | 8 |
| Other | 16 | 10 | 6 | 4 |  | 12 | 2 |  | 9 |

Note: Participants were able to report several gambling types.

EGM = Electronic Gambling Machine

SCI-GD = The Structured Clinical Interview for Gambling Disorder [(Grant et al., 2004)](https://www.zotero.org/google-docs/?IUm5Am)


Table 3

Internal consistency across samples

|  | Gambling cohort | | | |  | Gender | |  | Total |
| --- | --- | --- | --- | --- | --- | --- | --- | --- | --- |
| Gambling Disorder | Recreational  *n*=43 | Help-  seeking  *n*=79 | Self-help groups  *n*=31 | Treatment seeking  *n*=51 |  | Men  *n*=152 | Women  *n*=52 |  | Total  *N*=204 |
| Cronbach’s α^a^ |  |  |  |  |  |  |  |  |  |
| SCI-GD | 0.80 | 0.86 | 0.90 | 0.82 |  | 0.85 | 0.87 |  | 0.86 |
| Self-reported DSM-5 criteria | 0.80 | 0.90 | 0.82 | 0.70 |  | 0.86 | 0.85 |  | 0.86 |
| McDonald ω_h_ ^b^ |  |  |  |  |  |  |  |  |  |
| SCI-GD | 0.87 | 0.91 | 0.95 | 0.88 |  | 0.89 | 0.92 |  | 0.90 |
| Self-reported DSM-5 criteria | 0.86 | 0.93 | 0.90 | 0.83 |  | 0.90 | 0.90 |  | 0.90 |

Note:

^a^ = Raw alpha

^b^ = Total Omega

DSM-5 = the Diagnostic and Statistical Manual of Mental Disorders–fifth edition [(American Psychiatric Association, 2013)](https://www.zotero.org/google-docs/?XsqVaM)

SCI-GD = The Structured Clinical Interview for Gambling Disorder [(Grant et al., 2004)](https://www.zotero.org/google-docs/?NM490P)

Table 4

Convergent and discriminant validity across samples

|  | Gambling cohort | | | |  | Gender | |  | Total |
| --- | --- | --- | --- | --- | --- | --- | --- | --- | --- |
| Gambling Disorder | Recreational  *n*=43 | Help-  seeking  *n*=79 | Self-help groups  *n*=31 | Treatment seeking  *n*=51 |  | Men  *n*=152 | Women  *n*=52 |  | Total  *N*=204 |
| Convergent validity |  |  |  |  |  |  |  |  |  |
| PPGM |  |  |  |  |  |  |  |  |  |
| SCI-GD | 0.78 | 0.79 | 0.83 | 0.73 |  | 0.76 | 0.90 |  | 0.80 |
| Self-reported DSM-5 criteria | 0.80 | 0.92 | 0.31 | 0.61 |  | 0.77 | 0.74 |  | 0.77 |
| GDIT |  |  |  |  |  |  |  |  |  |
| SCI-GD | 0.67 | 0.76 | 0.71 | 0.66 |  | 0.71 | 0.80 |  | 0.75 |
| Self-reported DSM-5 criteria | 0.77 | 0.87 | 0.45 | 0.57 |  | 0.77 | 0.70 |  | 0.75 |
| Gambling debts |  |  |  |  |  |  |  |  |  |
| SCI-GD | 0.44 | 0.60 | -0.11 | 0.25 |  | 0.46 | 0.46 |  | 0.48 |
| Self-reported DSM-5 criteria | 0.50 | 0.64 | -0.08 | 0.10 |  | 0.57 | 0.55 |  | 0.57 |
| Discriminant validity |  |  |  |  |  |  |  |  |  |
| PHQ-9 |  |  |  |  |  |  |  |  |  |
| SCI-GD | 0.10 | 0.60 | 0.49 | 0.27 |  | 0.36 | 0.62 |  | 0.45 |
| Self-reported DSM-5 criteria | 0.80 | 0.92 | 0.31 | 0.61 |  | 0.77 | 0.74 |  | 0.77 |
| GAD-7 |  |  |  |  |  |  |  |  |  |
| SCI-GD | 0.07 | 0.60 | 0.34 | 0.33 |  | 0.32 | 0.58 |  | 0.41 |
| Self-reported DSM-5 criteria | 0.11 | 0.71 | 0.36 | 0.33 |  | 0.41 | 0.55 |  | 0.46 |

Note: Convergent and divergent validity was estimated using Pearson correlation between measure scores, having gambling debts (dichotomized), and number of fulfilled Gambling Disorder criteria, assessed via SCI-GD interviews or self-report.

DSM-5 = the Diagnostic and Statistical Manual of Mental Disorders–fifth edition [(American Psychiatric Association, 2013)](https://www.zotero.org/google-docs/?Bv60Yh)

GAD-7 = The Generalized Anxiety Disorder 7-item scale [(Spitzer et al., 2006)](https://www.zotero.org/google-docs/?L8jILR)

GD = Gambling Disorder [(American Psychiatric Association, 2013)](https://www.zotero.org/google-docs/?l9jueZ)

GDIT = The Gambling Disorder Identification Test [(Molander et al., 2021)](https://www.zotero.org/google-docs/?eYKRqU)

PHQ-9 = The Patient Health Questionnaire [(Kroenke et al., 2001)](https://www.zotero.org/google-docs/?jfhvF2)

PPGM = The Problem and Pathological Gambling Measure [(Williams & Volberg, 2013)](https://www.zotero.org/google-docs/?VKvQTg)

SCI-GD = The Structured Clinical Interview for Gambling Disorder [(Grant et al., 2004)](https://www.zotero.org/google-docs/?QKd22q)

Table 5

Rasch analysis of Gambling Disorder, assessed via SCI-GD interviews or self-reported DSM-5 criteria (*N* = 204)

|  |  | Item difficulty (SE) | | |  | Infit | |  | Outfit | |
| --- | --- | --- | --- | --- | --- | --- | --- | --- | --- | --- |
| DSM-5 criteria | | SCI-GD | Self-reported | Difficulty  delta^a^ |  | SCI-GD | Self-reported |  | SCI-GD | Self-reported |
| A1 | Tolerance | 1.20 (0.20) | 0.85 (0.18) | 0.35 |  | 1.11 | 1.11 |  | 1.01 | 1.16 |
| A2 | Abstinence | 0.60 (0.19) | -0.61 (0.19) | 1.21 |  | 1.17 | 0.82 |  | 1.30 | 0.67 |
| A3 | Loss of control | 0.11 (0.19) | -1.77 (0.22) | 1.88 |  | 0.93 | 0.83 |  | 0.97 | 0.86 |
| A4 | Preoccupation | -0.50 (0.20) | 0.68 (0.18) | -1.18 |  | 0.88 | 1.05 |  | 0.71 | 0.95 |
| A5 | Escape | 0.94 (0.19) | -0.79 (0.19) | 1.73 |  | 1.13 | 1.06 |  | 1.18 | 1.13 |
| A6 | Chasing losses | -0.39 (0.20) | -1.29 (0.20) | 0.90 |  | 0.72 | 0.71 |  | 0.50 | 0.50 |
| A7 | Lying | 0.41 (0.19) | -1.46 (0.21) | 1.87 |  | 0.85 | 0.90 |  | 0.71 | 0.83 |
| A8 | Jeopardized relationships, work and/or education | 0.79 (0.19) | 0.32 (0.18) | 0.47 |  | 1.06 | 1.25 |  | 1.10 | 1.25 |
| A9 | Relies financially on others | 1.28 (0.20) | 1.05 (0.19) | 0.23 |  | 0.98 | 1.09 |  | 0.93 | 1.08 |
| EAP reliability | | 0.84 | 0.83 |  |  |  |  |  |  |  |
| Underfit | |  |  | **>1.50** |  | 0 | 0 |  | 0 | 0 |
| Overfit | |  |  | **<0.50** |  | 0 | 0 |  | 0 | 0 |
| Person separation reliability  (*n* strata) | | 0.70 (2) | 0.72 (2) |  |  |  |  |  |  |  |

^a =^ Presented as item difficulty of the SCI-GD minus item difficulty of self-reported DSM-5 criteria.

DSM-5 = the Diagnostic and Statistical Manual of Mental Disorders–fifth edition [(American Psychiatric Association, 2013)](https://www.zotero.org/google-docs/?XsqVaM)

SCI-GD = The Structured Clinical Interview for Gambling Disorder [(Grant et al., 2004)](https://www.zotero.org/google-docs/?NM490P)

Table 6

Gambling Disorder, endorsed DSM-5 criteria by the SCI-GD diagnostic interview and self-report questionnaire (*N*=204)

| Gambling Disorder,  criteria and classification | | SCI-GD  *n* (%) | Self-reported  *n* (%) |
| --- | --- | --- | --- |
| A1 | Tolerance | 73 (36%) | 80 (39%) |
| A2 | Abstinence | 89 (44%) | 123 (60%) |
| A3 | Loss of control | 102 (50%) | 152 (75%) |
| A4 | Preoccupation | 118 (58%) | 85 (42%) |
| A5 | Escape | 80 (39%) | 128 (63%) |
| A6 | Chasing losses | 115 (56%) | 141 (69%) |
| A7 | Lying | 94 (46%) | 145 (71%) |
| A8 | Jeopardized relationships, work and/or education | 84 (41%) | 96 (47%) |
| A9 | Relies on others | 71 (35%) | 74 (36%) |
| Gambling Disorder, severity, *n* (%) | |  |  |
| No Gambling Disorder | | 94 (46%) | 58 (28%) |
| Any Gambling Disorder | | 110 (54%) | 146 (72%) |
| Mild Gambling Disorder | | 33 (16%) | 43 (21%) |
| Moderate Gambling Disorder | | 34 (17%) | 57 (28%) |
| Severe Gambling Disorder | | 43 (21%) | 46 (23%) |

DSM-5 = the Diagnostic and Statistical Manual of Mental Disorders–fifth edition [(American Psychiatric Association, 2013)](https://www.zotero.org/google-docs/?richsW)

SCI-GD = The Structured Clinical Interview for Gambling Disorder [(Grant et al., 2004)](https://www.zotero.org/google-docs/?xunARt)

Table 7

Comparisons between Gambling Disorder DSM-5 criteria (*N*=204)

|  | | SCI-GD vs self-reported DSM-5 criteria (*N*=204) | | | | |  |
| --- | --- | --- | --- | --- | --- | --- | --- |
| Gambling Disorder DSM-5 | | Correlations | |  | Kappa^a^ | |  |
|  |  | Pearson's *r* | Tetrachoric |  | Kappa | 95% CI |  |
| A1 | Tolerance | 0.41 | 0.60 |  | 0.40 | 0.27-0.54 |  |
| A2 | Abstinence | 0.43 | 0.65 |  | 0.39 | 0.25-0.53 |  |
| A3 | Loss of control | 0.49 | 0.80 |  | 0.40 | 0.26-0.53 |  |
| A4 | Preoccupation | 0.38 | 0.58 |  | 0.34 | 0.21-0.48 |  |
| A5 | Escape | 0.39 | 0.62 |  | 0.31 | 0.18-0.45 |  |
| A6 | Chasing losses | 0.55 | 0.78 |  | 0.52 | 0.38-0.65 |  |
| A7 | Lying | 0.50 | 0.80 |  | 0.40 | 0.27-0.54 |  |
| A8 | Jeopardized relationships, work and/or education | 0.35 | 0.52 |  | 0.34 | 0.21-0.48 |  |
| A9 | Relies on others | 0.45 | 0.66 |  | 0.44 | 0.32-0.59 |  |
| Symptom severity, Gambling Disorder | | - | - |  | 0.37 | 0.29-0.45 |  |

Note:

^a^ = Fleiss kappa for multiple raters and categorical variables [(Fleiss, 1971)](https://www.zotero.org/google-docs/?RB2abh)


DSM-5 = the Diagnostic and Statistical Manual of Mental Disorders–fifth edition [(American Psychiatric Association, 2013)](https://www.zotero.org/google-docs/?richsW)
