# Supplementary material for: Assessing Gambling Disorder Using Semistructured Interviews or Self-Report? Evaluation of the Structured Clinical Interview for Gambling Disorder Among Swedish Gamblers
Source: Assessment. 2023 Jan 21;30(8):2387–97. doi: 10.1177/10731911221147038 (PMC10623606; doi:10.1177/10731911221147038)
Supplement: sj-docx-3-asm-10.1177_10731911221147038 – Supplemental material for Assessing Gambling Disorder Using Semistructured Interviews or Self-Report? Evaluation of the Structured Clinical Interview for Gambling Disorder Among Swedish Gamblers [file sj-docx-3-asm-10.1177_10731911221147038.docx]

|  |  | | |  |
| --- | --- | --- | --- | --- |
|  | Initialer intervjuperson:_______________________ | Datum:_____________ | |  |
|  |  |  | |  |
|  | **HOPPA ÖVER OM REDAN KÄNT.** |  | |  |
|  |  |  |  |  |
|  | Har du någonsin spelat om pengar eller andra värdesaker? | ___Ja | ___ Nej |  |
|  |  |  |  |  |
|  |  | ANTECKNINGAR | |  |
|  | **OM ”JA”, FORTSÄTT:** |  | |  |
|  | Hur ofta spelar du om pengar nu? | ___________________ | |  |
|  | Hur mycket pengar spelar du för vanligtvis? | ___________________ | |  |
|  | När var senaste gången du spelade om pengar? | ___________________ | |  |
|  |  | ___________________ | |  |
|  | När spelade du som mest i ditt liv? | ___________________ | |  |
|  | Hur länge varade den perioden? | ___________________ | |  |
|  | Under den perioden …. | ___________________ | |  |
|  | hur ofta spelade du? | ___________________ | |  |
|  | vilken typ av spel spelade du? | ___________________ | |  |
|  | hur mycket pengar spelade du vanligtvis för? | ___________________ | |  |
|  |  | ___________________ | |  |
|  | Under den perioden …. | ___________________ | |  |
|  | skapade ditt spelande problem för dig? | ___________________ | |  |
|  | reagerade någon på att du spelade? | ___________________ | |  |
|  |  | ___________________ | |  |
|  |  | ___________________ | |  |
|  |  | ___________________ | |  |

**FORTSÄTT OM DET FINNS INDIKATION PÅ HÖG FREKVENS AV SPELANDE ELLER SPEL FÖR HÖGA SUMMOR ELLER NEGATIVA SOCIALA, JURIDISKA, FAMILJE-, SKOL- ELLER YRKESMÄSSIGA KONSEKVENSER PÅ GRUND AV SPEL.**

| INFORMATION TILL INTERVJUARE:  NOTERA SVAREN FÖR VARJE FRÅGA I  UTRYMMET NEDAN. |  | För varje kriterium, ringa in ett av  följande fyra svar:  ? = Otillräcklig information  1 = Föreligger ej/falskt  2 = Ej fullt säkerställt  3 = Säkerställt/sant | |  |
| --- | --- | --- | --- | --- |
| A Kriterium |  | Ihållande och upprepat problematiskt spel om pengar som visar sig i minst 4 av följande 9 kriterier nedan under en 12-månadersperiod: |  |  |
| Jag skulle vilja ställa lite fler frågor angående ditt spelande. Vi kommer att fokusera på den tidsperiod då du spelade som mest, men också hur det sett ut det senaste året. |  |  | |  |
|  |  |  | |  |
| **INFORMATION TILL INTERVJUARE:**  **ÄNDRA TEMPUS I FRÅGORNA OM MESTA SPELANDET LIGGER LÄNGRE TILLBAKA I TIDEN.** |  | Kriterierna för Hasardspelsyndrom står i annan ordning än i DSM-5. | Kriterium  bedömning | |
| Hur ofta tänker du på spel?  Hur mycket tänker du på tidigare speltillfällen?  Hur ofta föreställer du dig eller planerar  framtida speltillfällen?  Hur ofta tänker du på hur du ska få tag på pengar för att spela eller betala spelskulder?  Hindrar tankar på spel dig från att fokusera på arbetet, familjen eller andra viktiga  ansvarsområden?  Hur har det sett ut för dig de senaste 12 månaderna? (om denna period inte infallit de senaste 12 månaderna) |  | **A4.** Är ofta upptagen av hasardspel (ex ihållande tankar kring tidigare speltillfällen, analyserar och planerar för kommande speltillfällen, tänker ut olika sätt att få pengar att spela med). | Senaste 12  månaderna  ?  1  2  3 | |
|  |  |  |  |  |
| Under den period som du spelade som mest, vilka var anledningarna till att du spelade?  **OM INFORMATION INTE FRAMKOMMER, FRÅGA:**  Spelade du någonsin för att…  fly från problem i ditt liv?  minska obehagliga känslor eller dåligt   mående?  Hur ofta hände det?  Hur har det sett ut för dig de senaste 12 månaderna? (om denna period inte infallit de senaste 12 månaderna) |  | **A5.** Spelar ofta när hen är plågad av negativa känslor (tex hjälplöshet, skuld, ångest, nedstämdhet). | Senaste 12  månaderna  ?  1  2  3 | |
| Förra frågan berättade du att du spelade för att ____________________________________  ____________________________________  Har du behövt öka summan satsade pengar, för att få önskad effekt av spelandet?  OM ”JA”: Hur mycket ökade summorna?  OM ”NEJ”: Upplevde du mycket mindre effekt  av spelandet då du spelade för  samma summa?  Hur har det sett ut för dig de senaste 12  månaderna? |  | **A1.** Behöver spela med allt större summor pengar för att uppnå den önskade spänningseffekten.  (Tolerans definieras som ett av följande: (a) ett behov av markant ökade insatser för att uppnå samma effekt, (b) markant minskad effekt för hasardspel med samma insatser). | Senaste 12  månaderna  ?  1  2  3 | |
| När du har förlorat, har du någonsin “jagat dina förluster”? Alltså har du spelat för att försöka “bli kvitt”?  OM ”NEJ”: När du har förlorat, har du spelat   för större summor för att försöka  kompensera för förlusten?  Hur har det sett ut för dig de senaste 12  månaderna? |  | **A6.** Återvänder ofta en annan dag för att revanschera sig efter att ha spelat bort pengar (”jagar” förlusterna).  (Stora förluster leder till en ökning av frekvens och storlek av satsade summor. Mönstret förekommer under ett enskilt spelskov en eller flera dagar, eller under flera enskilda dagar spridda över en tidsperiod). | Senaste 12  månaderna  ?  1  2  3 | |
| Har du någonsin ljugit för någon om ditt spelande, exempelvis hur lång tid du spelat, för vilka summor, eller att du spelat  överhuvudtaget?  För vem har du ljugit?  Hur ofta?  Hur har det sett ut för dig de senaste 12 månaderna? |  | **A7.** Ljuger för att dölja omfattningen av sitt spelande.  (Ljuger för familje-  medlemmar, vårdpersonal eller signifikanta andra för att dölja omfattningen av spelandet. Mönster av lögner under lång tid med syfte att kunna fortsätta spela utan att bli avbruten eller störd). | Senaste 12  månaderna  ?  1  2  3 | |
| Har ditt spelande skapat problem för dig vad gäller familjeliv, jobb, skola eller sociala relationer… Till den utsträckning att du riskerat att förlora en viktig relation eller något annat  betydelsefullt?  Har ditt spelande lett till andra negativa konsekvenser, exempelvis dåligt rykte eller  försämrad psykisk och/eller fysisk hälsa?  Hur har det sett ut för dig de senaste 12  månaderna? |  | **A8.** Har äventyrat eller förlorat någon viktig  personlig relation,  anställning, utbildnings- eller karriärmöjlighet på grund av spelandet. | Senaste 12  månaderna  ?  1  2  3 | |
|  | | | |  |
| Har du försökt kontrollera, begränsa eller  sluta helt med ditt spelande?  OM ”JA”: Hur många gånger?  Hur har det gått? Har du lyckats begränsa ditt spelande eller sluta spela?  Har du någonsin lyckats sluta spela helt?  OM ”NEJ”: Har du någonsin velat sluta eller  dra ned på spelandet?  OM ”JA”: Är ditt spelande någonting du oroat  dig över?  Hur har det sett ut för dig de senaste 12  månaderna? |  | **A3.** Har gjort upprepade misslyckande ansträngningar att kontrollera, begränsa eller upphöra med hasardspel.  (Ihållande önskan att kontrollera, begränsa, dra ned på eller sluta spela; eller misslyckade försök till detta.) | Senaste 12  månaderna  ?  1  2  3 | |
| OM ”JA” PÅ FÖRSÖK ATT KONTROLLERA, BEGRÄNSA ELLER SLUTA SPELA, FRÅGA: Upplevde du rastlöshet eller irritabilitet när du försökte begränsa eller sluta med ditt spelande?  Upplevde du obehag eller kände dig upprörd när du upphörde med ditt spel, såsom: irritation, sömnsvårigheter, svettningar, darrande händer eller ångest?  OM ”NEJ” TILL OVANSTÅENDE SYMTOM VID FÖRSÖK ATT KONTROLLERA, BEGRÄNSA ELLER SLUTA SPELA, FRÅGA: Har du upplevt några av sakerna vi pratade om alldeles nyss då du ville spela men det inte gick… exempelvis att du inte hade tillgång till pengar eller att det inte gick att spela i just den situationen?  Hur har det sett ut för dig de senaste 12  månaderna? |  | **A2.** Är rastlös eller irritabel vid försök att begränsa eller upphöra med hasardspel.  (Minst två av följande abstinenssymtom: ångest, irritabilitet, rastlöshet, sömnsvårigheter,  svettningar, darrande  händer) | Senaste 12  månaderna  ?  1  2  3 | |
| Har du någonsin bett någon familjemedlem eller nära vän om pengar, för att kunna lösa en finansiell krissituation du hamnat i på grund av spel?  Hur har det sett ut för dig de senaste 12  månaderna? |  | **A9.** Förlitar sig på att andra kan ordna fram pengar för att lösa finansiella krissituationer som uppstår på grund av spelandet.  (Närstående räddar personen ur finansiella krissituationer genom att låna ut eller ge bort pengar.) | Senaste 12  månaderna  ?  1  2  3 | |

**B Kriterium.**

| **INFORMATION TILL INTERVJUARE:**  **OM SCI-GD ANVÄNDS TILLSAMMANS MED SCID, HÄNVISA TILL KRITERIER FÖR**  **MANISK EPISOD.**  **OM KRITERIER FÖR MANISK EPISOD EJ ÄR UPPFYLLT, BEDÖMS SOM ”SPELBETEENDE FÖRKLARAS INTE BÄTTRE MED MANISK EPISOD”.**  **OM KRITERIET FÖR MANISK EPISOD ÄR UPPFYLLT, FRÅGA:**  Spelar du enbart under de perioder du upplever _______________________________________ **(ANVÄND ORD FÖR ATT BESKRIVA MANI)**?  Spelar du oftast bara då är _________________________ **(BESKRIV MANISKA SYMTOM)**  exempelvis:  …. sover enbart några få timmar per natt och känner  sig ändå utvilad?  … känner sig mer självsäker än vanligt?  … upplever att tankarna rusar genom huvudet?  … upplever svårigheter att bibehålla koncentration  och fokus.  **PÅMINNELSE TILL INTERVJUARE: PERIOD AV MANISKT BETEENDE MÅSTE UPPGÅ TILL MINST EN VECKA FÖR ATT KVALIFICERAS SOM MANISK EPISOD.** |  | **B.** Spelbeteendet förklaras inte bättre med manisk episod.  **INFORMATION TILL INTERVJUARE: SKATTA 3 FÖR SPELBETEENDE SOM INTE FÖRKLARAS BÄTTRE NED MANISK EPISOD.**  (Då det inträffar att personer under en manisk episod spelar på samma vis som vid Hasardspelssyndrom, måste Manisk episod uteslutas som primär diagnos. Det är dock möjligt att ställa både Hasardspelsyndrom och Bipolär typ 1 diagnos. | ?  1  2  3 |
| --- | --- | --- | --- |
|  |  |  |  |

|  | **HASARDSPESYNDROM** | |  |  |  |  |  |
| --- | --- | --- | --- | --- | --- | --- | --- |
|  |  |  | |  |  |  |  |
|  | **Antal kriterier**  **uppfyllda:** | _________ | |  |  |  |  |
|  | **B kriterie uppfyllt:** | Ja ____ | Nej ____ |  |  |  |  |
|  |  |  |  |  |  |  |  |
|  | **FÖR ATT UPPFYLLA DIAGNOS MÅSTE MINST**  **4 AV 9 ”A” KRITERIER UPPFYLLAS OCH ”B”**  **KRITERIET, UNDER SAMMA 12**  **MÅNADERSPERIOD.** | | |  | ***HASARDSPELSYNDROM***  **NEJ JA**  **SENASTE 12 MÅNADERNA** | | |
|  |  | | | |  |  |  |
|  |  |  |  |  |  | | |
|  | DEFINITIONER FÖR HASARDSPELSYNDROM: | | |  | **ANGE OM:** |  |  |
|  | Lindrigt: 4-5 kriterier är uppfyllda. | | |  | **LINDRIGT** | ☐ |  |
|  | Medelsvårt: 6-7 kriterier är uppfyllda. | | |  | **MEDELSVÅRT** | ☐ |  |
|  | Svårt: 8-9 kriterier är uppfyllda. | | |  | **SVÅRT** | ☐ |  |
|  |  | | |  |  |  |  |
|  | Episodiskt: Uppfyller under mer än en period kriterierna för hasardspelsyndrom. Mellan dessa perioder avtar symtomen under åtminstone några månaders tid. | | |  | **EPISODISKT** | ☐ |  |
|  | Ihållande: Kontinuerliga symptom som uppfyller kriterierna under flera års tid. | | |  | **IHÅLLANDE** | ☐ |  |
|  |  | | |  |  |  |  |
|  | I tidig remission: Kriterierna för hasardspelsyndrom har tidigare varit helt uppfyllda. Under minst 3 månaders tid, men kortare än 12 månader, har inga kriterier varit uppfyllda. | | |  | **I TIDIG REMISSION** | | ☐ |
|  | I varaktig remission: Kriterierna för hasardspelsyndrom har tidigare varit helt uppfyllda. Under minst 12 månaders tid  har inga kriterier varit uppfyllda. | | |  | **I VARAKTIG**  **REMISSION** | | ☐ |

**Om svenska Structured Clinical Interview for Gambling Disorder (SCI-GD)**

Structured Clinical Interview for Gambling Disorder (SCI-GD) är en semistrukturerad intervju för att fastställa symptom på hasardspelsyndrom (på engelska Gambling Disorder, 312.31 enligt DSM 5). Intervjun är fri att använda i forskning och klinisk verksamhet (ej för kommersiellt bruk).

Svenska SCI-GD är en godkänd översättning av den amerikanska versionen av Structured Clinical Interview for Pathological Gambling (SCI-PG, senare benämnd SCI-GD i och med namnbytet till Gambling Disorder i DSM-5). SCI-GD följer samma struktur som Structured Clinical Interview for DSM-IV Disorders (SCID), som är ett vanligt förekommande instrument inom psykiatrisk

diagnostik. Den intervjuade får beskriva med egna ord istället för att bekräfta eller dementera ett påstående. Intervjuaren ställer uppföljande frågor tills det kan säkerställas om ett kriterium varit uppfyllt eller inte.

**Kommentarer till svenska SCI-GD**

Till skillnad från engelska originalversionen inkluderar svenska SCI-GD även bedömning av

hasardspelsyndrom det senaste året, och inte bara den svåraste tidigare episoden av spelande.

I enlighet med diagnosen används **B** ”spelar endast under maniska skov” som exklusionskriterium. Observera att en person samtidigt kan uppfylla kriterier för bipolärt syndrom och

hasardspelsyndrom. Om personen uppger sig spela *uteslutande* under maniska skov, kan ej

diagnosen hasardspelsyndrom ställas.

Under **A9** efterfrågas huruvida personen har lånat pengar av vänner eller familj för att hantera en finansiell kris som uppstod av spelandet. Det bör påpekas att det i en svensk kontext är vanligt

förekommande att personer med spelproblem tar olika snabblån för att hantera ekonomiska problem och att detta kan bedömas likvärdigt som ett lån från en anhörig.

**Referenser**

Grant, J. E., Steinberg, M. A., Kim, S. W., Rounsaville, B. J., & Potenza, M. N. (2004). Preliminary validity and reliability testing of a structured clinical interview for pathological gambling.

*Psychiatry research*, *128*(1), 79-88.
